# Intestinal vitamin D receptor protects against extraintestinal breast cancer tumorigenesis

**DOI:** 10.1080/19490976.2023.2202593

**Published:** 2023-04-19

**Authors:** Yong-Guo Zhang, Yinglin Xia, Jilei Zhang, Shreya Deb, Shari Garrett, Jun Sun

**Affiliations:** a Division of Gastroenterology and Hepatology, Department of Medicine, University of Illinois Chicago, Chicago, IL, USA; b Department of Microbiology and Immunology, University of Illinois Chicago, Chicago, IL, USA; c UIC Cancer Center, University of Illinois Chicago, Chicago, IL, USA; d Jesse Brown VA Medical Center Chicago, Chicago, IL, USA

**Keywords:** Dysbiosis, breast cancer, barrier function, butyrate-producing bacteria, butyrate, inflammation, gut-breast-axis, *Lactobacillus plantarum*, probiotics, tight junctions, VDR

## Abstract

The microbiota plays critical roles in regulating the function and health of the intestine and extraintestinal organs. A fundamental question is whether an intestinal-microbiome-breast axis exists during the development of breast cancer. If so, what are the roles of host factors? Vitamin D receptor (VDR) involves host factors and the human microbiome. Vdr gene variation shapes the human microbiome, and VDR deficiency leads to dysbiosis. We hypothesized that intestinal VDR protects hosts against tumorigenesis in the breast. We examined a 7,12-dimethylbenzanthracene (DMBA)-induced breast cancer model in intestinal epithelial VDR knockout (VDR^ΔIEC^) mice with dysbiosis. We reported that VDR^ΔIEC^ mice with dysbiosis are more susceptible to breast cancer induced by DMBA. Intestinal and breast microbiota analysis showed that VDR deficiency leads to a bacterial profile shift from normal to susceptible to carcinogenesis. We found enhanced bacterial staining within breast tumors. At the molecular and cellular levels, we identified the mechanisms by which intestinal epithelial VDR deficiency led to increased gut permeability, disrupted tight junctions, microbial translocation, and enhanced inflammation, thus increasing tumor size and number in the breast. Furthermore, treatment with the beneficial bacterial metabolite butyrate or the probiotic Lactobacillus plantarum reduced breast tumors, enhanced tight junctions, inhibited inflammation, increased butyryl-CoA transferase, and decreased levels of breast Streptococcus bacteria in VDR^ΔIEC^ mice. The gut microbiome contributes to the pathogenesis of diseases not only in the intestine but also in the breast. Our study provides insights into the mechanism by which intestinal VDR dysfunction and gut dysbiosis lead to a high risk of extraintestinal tumorigenesis. Gut-tumor-microbiome interactions represent a new target in the prevention and treatment of breast cancer.

## Introduction

Vitamin D is a group of fat-soluble steroids responsible for multiple biological effects. The active form of vitamin D, in conjunction with its own receptor VDR, exerts important roles in modulating both mucosal immunity and normal growth of epithelia cells ^1, 2^. The dysregulation of the vitamin D/VDR is known to increase the risk of various human disorders^3–5^, including breast cancer^6–9^. The parallel appreciation of a role for VDR in cancer biology began approximately 3 decades ago, and an understanding of its actions in normal and malignant systems has subsequently increased^10^.

The VDR-dependent regulation of the gut microbiome in human and animal studies represents a newly identified and highly significant role for VDR^11,12^. We have demonstrated that the variations in the human *Vdr* gene shape the gut microbiome and that VDR deletion leads to dysbiosis^12^. Our studies support the critical role of VDR in maintaining intestinal and microbial homeostasis^13–15^. We established the first conditional deletion of intestinal epithelial VDR mouse model (VDR^ΔIEC^) and demonstrated that intestinal bacterial abundance and function are significantly altered in VDR^ΔIEC^ mice^11,16^. VDR^ΔIEC^ mice were also susceptible to inflammatory triggers^11^ and colonic tumorigenesis^15^, indicating that intestinal VDR contributes to host protection against injury and inflammation. Dysbiosis and chronic inflammation are important contributors to the development of cancer.

Research progress on the VDR microbiome has established a microorganism-induced program of epithelial cell homeostasis and repair in the intestine^17^. Dysregulation of bacterial-host interactions can result in chronic inflammatory and overexuberant repair responses in the development of cancer^18–23^. Polymorphisms of the *vdr* gene (Bsm1, Apa1, Fok1, and Poly(A)) were reported to increase susceptibility to breast cancer^24,25^. VDR expression level in breast tumor tissue microarrays has shown inversely associated with aggressiveness of breast cancer, but not with cancer survival outcomes^26^. Although vitamin D/VDR is an active topic in cancer research^14,15,26–29^, the mechanism underlying host-microbiome interactions in tumorigenesis is incompletely understood. We know little about the effects and mechanisms by which intestinal epithelial VDR and the microbiome influence dysbiosis and the development of breast cancer.

In the current study, we hypothesized that intestinal VDR protects hosts against tumorigenesis in the breast. We found that VDR^ΔIEC^ mice with intestinal dysbiosis are more susceptible to breast cancer induced by DMBA. Intestinal and breast microbiota analysis showed that VDR deficiency leads to a bacterial profile shift from normal to one that is susceptible to carcinogenesis. At the cellular level, we identified the mechanisms by which intestinal epithelial VDR deficiency led to increased gut permeability, disrupted tight junctions, microbial translocation, and enhanced inflammation, thus increasing tumor size and number in the breast. Furthermore, treatment with the beneficial bacterial metabolite butyrate or a probiotic *Lactobacillus plantarum* strain reduced breast tumors in VDR^ΔIEC^ mice. Our study provides new insights into the mechanism by which intestinal VDR dysfunction leads to a high risk of extraintestinal tumorigenesis in the breast.

## Results

### Altered bacterial diversity and risk of cancer in intestinal VDR-deficient mice

We first checked whether intestinal epithelial VDR deletion has any effects on the microbiome and the risk of breast cancer. We performed metagenomic sequencing of 20 fecal samples from 2 groups, namely, the VDR^ΔIEC^ mouse group, which has conditional deletion of VDR in intestinal epithelial cells (5 males and 5 females), and the control group of VDR^loxp^ mice (3 males and 7 females). After removing the repeated sequence with≥99% identity, 52892,651 taxonomic alignments with an average of 2,644,633 reads per sample and 27,893,990 functional alignments with an average of 1,394,700 reads per sample were generated.

**Figure 1.**
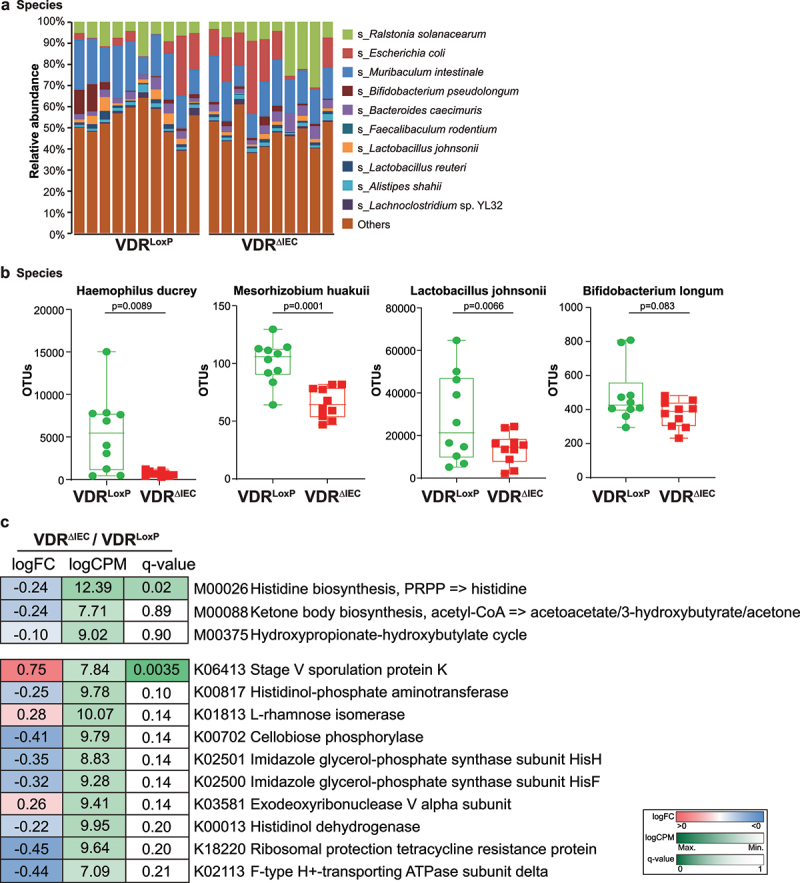
Altered taxonomic community of intestinal bacteria in VDR^ΔIEC^ mice compared with VDR^loxp^ mice. (a) Relative bacterial abundances at the species level are shown for the top 10 species, and less abundant species were grouped as “others”. Each bar represents an individual mouse, n = 10 per group. (b) the presentive bacterial species that were markedly altered after intestinal VDR conditional deletion. The values of the Y-axis are based on operational taxonomic unit (OTU) counts, representing the sequence reads. Data are expressed as the mean ± SD, Welch’s t test, n = 10 each group. (c) Differential analysis of functional genes in the feces of conditional VDR-knockout mice. The KEGG MODULE database consists of KEGG modules identified by M numbers, which are manually defined functional units of gene sets. The KEGG Module ortholog table is a useful tool to check the completeness and consistency of genome annotations. It shows currently annotated genes in individual genomes for a given set of K numbers. Items with q-values≤0.05 in pairwise comparisons or butyrate-related items were selected. The fold-change (log_2_FC), counts per million (log_2_CPM), and q-value were colored using the key as indicated on the right side of the figure, n = 10 each group. All *p* values are shown in the figures.

As shown in Figure 1a, the bacterial community profiles showed differences in the diversity and composition of the studied animals between the control group VDR^loxp^ mice and VDR^ΔIEC^ mice. The top 10 most prevalent bacterial species in each animal present individually were *Ralstonia solanacearum*, *Escherichia coli*, *Muribaculum intestinale*, *Bifidobacterium pseudolongum*, *Bacteroides caecimuris*, *Faecalibaculum rodentium*, *Lactobacillus johnsonii*, *Lactobacillus reuteri*, *Alistipes shahii*, and *Lachnoclostridium* sp. YL32 (Figure 1a). *Haemophilus ducreyi* is a gram-negative bacterium and causative agent of genital ulcer disease chancroid^30^ and was found to be significantly downregulated in VDR^ΔIEC^ mice (Figure 1b). *Mesorhizobiu*m *huakuii* induces the formation of nitrogen-fixation nodules on its host plant *Astragalus sinicus* and has been assigned to a new biovariant^31^. *M. huakuii* isolates were also found to have endotoxic activity against lipopolysaccharides^32^ and were significantly downregulated in our VDR^ΔIEC^ mice. Moreover, two beneficial bacterial species, *Lactobacillus johnsonii* and *Bifidobacterium pseudolongum*, were markedly downregulated in VDR^ΔIEC^ mice (Figure 1b).

In addition to bacterial diversity and abundance, shotgun metagenomic sequencing could reveal microbial functional alterations through functional analysis. We performed taxonomic functional module, pathway, and table analyses, which were mainly based on the set of related biosynthesis, individual pathway, and functional genes, respectively. Then, we performed differential analysis on the functional profiling to show the impacts of VDR conditional deletion on these identified functions in modules, pathways, and tables. Interestingly, some butyrate-related modules, e.g., the hydroxypropionate-hydroxybutylate cycle and ketone body biosynthesis (acetyl-CoA => acetoacetate/3-hydroxybutyrate/acetone), were downregulated in the VDR^ΔIEC^ mice, and the gene encoding stage V sporulation protein K, which is essential for sporulation and is specific to stage V sporulation^33^, was significantly (q < 0.01) upregulated in the VDR^ΔIEC^ mice (Figure 1c). The histidine biosynthesis pathway, which is an ancient metabolic pathway present in bacteria, archaea, lower eukaryotes and plants and has fundamental regulatory processes in bacteria (e.g., proton buffering and metal ion chelation)^34,35^, was found to be significantly (q < 0.05) downregulated in VDR^ΔIEC^ mice but upregulated in other pairwise comparisons without any significant difference (Figure 1c).

## VDR^ΔIEC^ mice developed more and larger breast tumors than VDR^loxp^ mice

**Figure 2.**
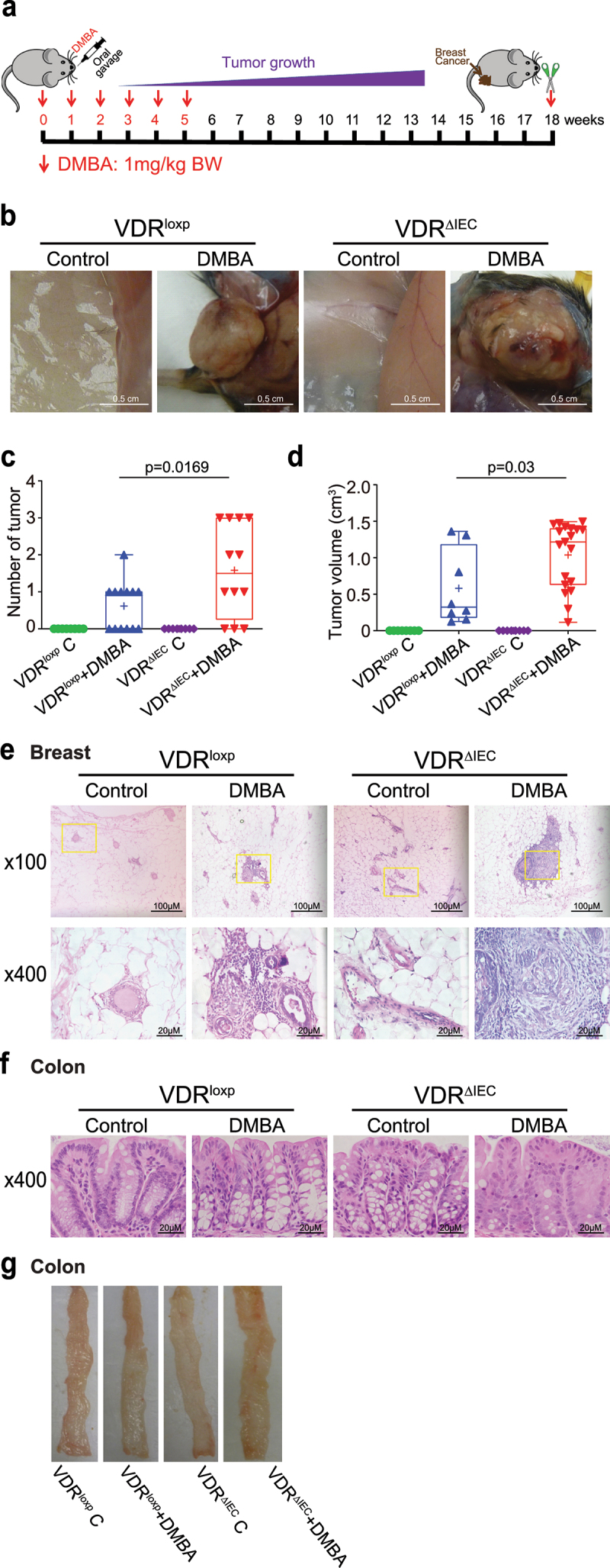
VDR^ΔIEC^ mice developed larger and more breast tumors. (a) Schematic overview of the DMBA-induced breast cancer model. Mice were given 1.0 mg of DMBA in 0.2 ml of corn oil by oral gavage once a week for 6 weeks. The samples were harvested at week 18. (b) Breast tumors in situ. Representative mammary glands from different groups. (c) the number of breast tumors significantly increased in VDR^ΔIEC^ mice compared with VDR^loxp^ mice. Data are expressed as the mean ± SD. N = 8–13, one-way ANOVA. (d) the breast tumor volumes were significantly larger in VDR^ΔIEC^ mice than in VDR^loxp^ mice. Data are expressed as the mean ± SD. n = 8–13, one-way ANOVA. (e) Representative H&E staining of mammary glands from the indicated groups. Images were from a single experiment and are representative of 8–13 mice per group. (f) Representative H&E staining of intestines from the indicated groups. The images were from a single experiment and are representative of 8–13 mice per group. All *p* values are shown in the figures. (g) Representative photographs of colons from the indicated groups.

Gut dysbiosis is associated with the development of breast cancer^36–40^. Our metagenomic sequencing results indicated that intestinal epithelial VDR knockout induced gut microbiome dysbiosis. We then investigated the role of intestinal VDR in the development of breast cancer using a DMBA mouse model (Figure 2a). Chemically induced rodent models of breast cancer have been extensively used to reflect the initiation and progression of human breast cancer^41–44^. We found a striking difference in breast tumor incidence between VDR^loxp^ and VDR^ΔIEC^ mice treated with DMBA. Representative breast tumors are shown in Figure 2b. The number of breast tumors was significantly increased in VDR^ΔIEC^ mice compared with VDR^loxp^ mice (Figure 2c). The volumes of the tumors were significantly larger in the VDR^ΔIEC^ mice than in the VDR^loxp^ mice (Figure 2d). Furthermore, pathological analysis of breast samples indicated tumor size differences between the VDR^loxp^ and VDR^ΔIEC^ mouse DMBA experimental groups (Figure 2e). However, by H&E, the colon and ileum did not show tumors in VDR^loxp^ and VDR^ΔIEC^ mice treated with DMBA (Figure 2(f–g) Figure S1).

## Intestinal epithelial VDR deletion led to decreased VDR expression, increased proliferation, and decreased apoptosis in breast tumor tissues

**Figure 3.**
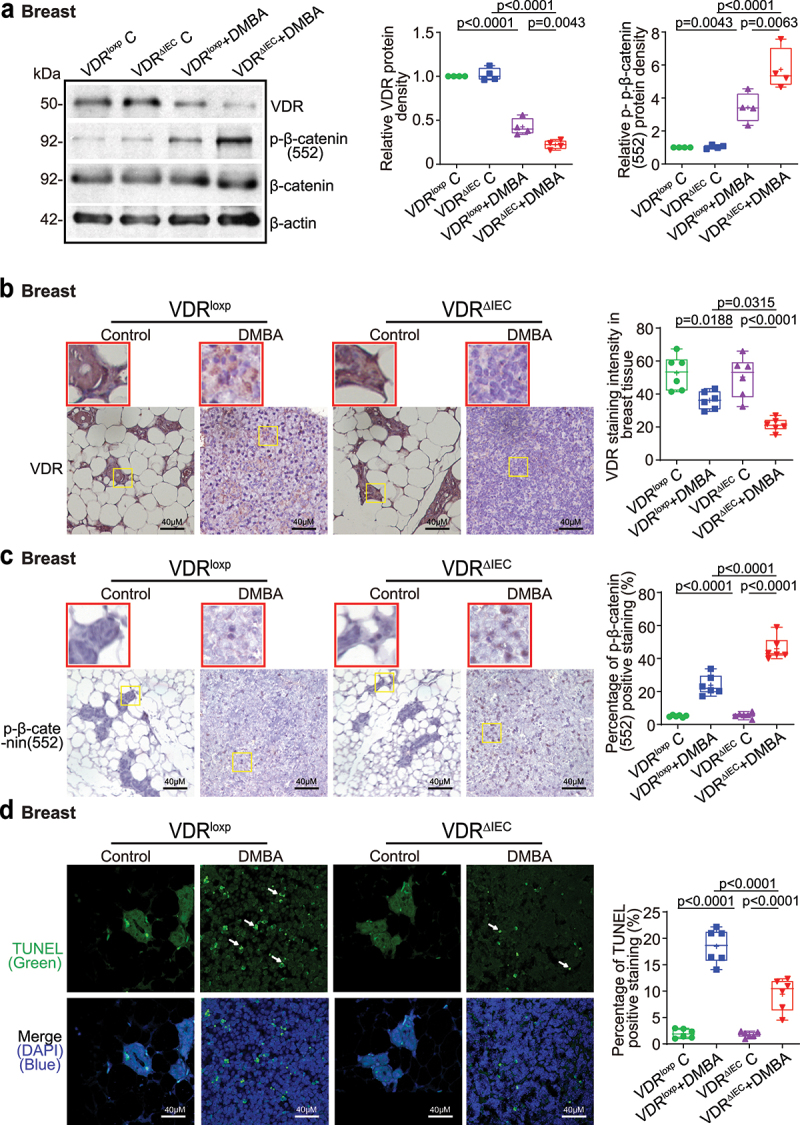
Intestinal epithelial VDR deletion led to decreased VDR expression, increased proliferation, and decreased apoptosis in breast tumor tissues. (a) Decreased VDR protein expression and increased *p*-β-catenin (Ser552) expression in mammary gland tumors of VDR^ΔIEC^ mice compared with VDR^loxp^ mice. Data are expressed as the mean ± SD. N = 4, one-way ANOVA. (b) VDR was decreased in breast tumors of VDR^ΔIEC^ mice compared with VDR^loxp^ mice by IHC staining. Images are from a single experiment and are representative of 6 mice per group. Red boxes indicate the selected area at higher magnification. (c) *p*-β-catenin (Ser552) expression increased in breast tissues of VDR^ΔIEC^ mice compared with VDR^loxp^ mice by IHC staining. Images are from a single experiment and are representative of 6 mice per group. Red boxes indicate the selected area at higher magnification. (d) Apoptosis-positive cells were decreased in the breast tissue of VDR^ΔIEC^ mice compared with VDR^loxp^ mice by TUNEL staining. Images are from a single experiment and are representative of 6 mice per group. All *p* values are shown in the figures.

In VDR^ΔIEC^ mice, we observed significant downregulation of VDR at the protein level in breast tumor tissue (Figure 3a). Reduced VDR expression was confirmed via IHC staining of breast tissues of the control and DMBA-treated VDR^loxp^ and VDR^ΔIEC^ mice (Figure 3b). Our WB and IHC data of the proliferative marker *p*-β-catenin (ser 552)^45–47^ showed that *p*-β-catenin (Ser552) in breast tumor tissue was significantly increased in VDR^ΔIEC^ mice compared to VDR^loxp^ mice (Figure 3a–c). Apoptosis-positive cells were decreased in the breast tumor tissue of VDR^ΔIEC^ mice compared with VDR^loxp^ mice by TUNEL staining (Figure 3d). Altered cell proliferation and apoptosis in the breasts of VDR^ΔIEC^ mice ultimately enhanced their susceptibility to carcinogenesis.

## Intestinal VDR deletion led to increased gut permeability, disrupted tight junctions, microbial translocation, and enhanced inflammation

**Figure 4.**
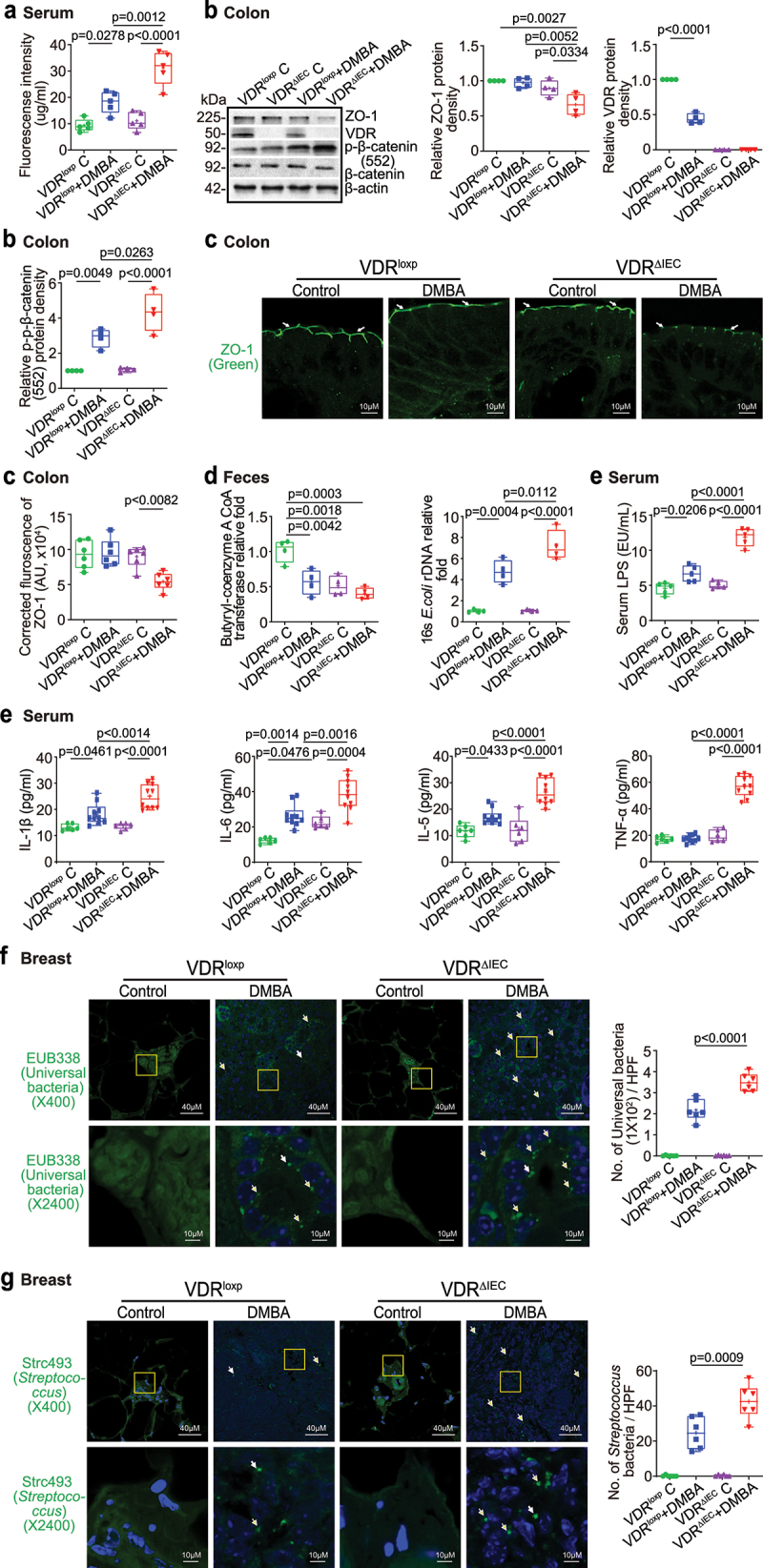
Increased intestinal permeability, decreased ZO-1 expression, chronic inflammation and increased universal bacteria in breast tumors of VDR^ΔIEC^ mice compared with VDR^loxp^ mice. (a) Intestinal permeability increased in the DMBA-induced VDR^ΔIEC^ breast cancer model. Fluorescein dextran (molecular weight 4 kDa, diluted in HBSS) was gavaged (50 mg/kg mouse). Four hours later, mouse blood samples were collected for fluorescence intensity measurement. Data are expressed as the mean ± SD. N = 5, one-way ANOVA. (b) ZO-1 expression decreased in the intestine of VDR^ΔIEC^ mice after DMBA treatment compared with VDR^loxp^ mice. The expression of *p*-β-catenin (552) increased in the colon of VDR^ΔIEC^ mice after DMBA treatment compared with VDR^loxp^ mice. Data are expressed as the mean ± SD; n = 4, one-way ANOVA. (c) ZO-1 expression decreased in intestinal VDR^ΔIEC^ mice after DMBA treatment compared with VDR^loxp^ mice by IF staining. Images are from a single experiment and are representative of 6 mice per group. Data are expressed as the mean ± SD. N = 6, one-way ANOVA. (d) Lack of intestinal VDR led to dysbiosis and a shift in the bacterial profile. Expression of butyryl-coenzyme a CoA transferase decreased in control tissue and in tumors in VDR^ΔIEC^ mice compared to VDR^loxp^ mice. *E.* *coli* was enhanced in tumors in VDR^ΔIEC^ mice compared to VDR^loxp^ mice. Data are expressed as the mean ± SD. N = 4, one-way ANOVA. (e) Serum LPS, IL-1β, IL-6, IL-5, and TNF-α were significantly higher in tumors in VDR^ΔIEC^ mice than in VDR^loxp^ mice. Serum samples were collected from VDR^loxp^ and VDR^ΔIEC^ mice with or without tumors, and cytokines were detected by a Luminex detection system. Data are expressed as the mean ± SD. N = 6–10, one-way ANOVA. (f) More universal bacteria in the breast tumor tissue of VDR^ΔIEC^ mice were found by fluorescence in situ hybridization. Images are from a single experiment and are representative of 6 mice per group. Data are expressed as the mean ± SD. N = 6, one-way ANOVA. All *p* values are shown in the figures. (g) More *Streptococcus* bacteria in the breast tumor tissue of VDR^ΔIEC^ mice were found by fluorescence in situ hybridization. Images are from a single experiment and are representative of 6 mice per group. Data are expressed as the mean ± SD. N = 6, one-way ANOVA. All *p* values are shown in the figures.

Gut dysbiosis usually increases harmful intestinal bacteria, which may release more enterotoxins, e.g., LPS, damaging tight junctions (TJs) in epithelial cells and thereby increasing the permeability of the intestine and elevating the risk of cancer^36,48^. To test intestinal permeability, mice were gavaged with fluorescein dextran 18 weeks after the first DMBA treatment. Four hours later, blood samples were collected for fluorescence intensity measurement. Higher fluorescence intensity indicated increased intestinal permeability. As shown in Figure 4a, DMBA treatment increased intestinal permeability in both VDR^loxp^ and VDR^ΔIEC^ mice, while VDR^ΔIEC^ mice exhibited significantly higher permeability post-treatment. Based on the *in vivo* intestinal permeability data, we hypothesized that TJ proteins might be altered in DMBA-treated mice. In the VDR^ΔIEC^ mice, we observed significant downregulation of ZO-1 at the protein level in the colon, while *p*-β-catenin (552) increased at the protein level in the colon (Figure 4b). Reduced and disorganized ZO-1 was confirmed by immunostaining of the colon in DMBA-treated mice (Figure 4c). Butyrate synthesis by anaerobic bacteria can occur via butyryl-coenzyme A (CoA):acetate CoA-transferase^49^. We found that intestinal VDR deficiency led to dysbiosis and a shift in the bacterial profile. The expression of butyryl-CoA transferase decreased in the feces of VDR^ΔIEC^ mice compared to VDR^loxp^ mice. *E. coli* was enhanced in VDR^ΔIEC^ mice compared to VDR^loxp^ mice (Figure 4d). Chronic inflammation is a key factor that contributes to breast cancer. We found that serum LPS and the cytokines IL-1β, IL-6, IL-5 and TNF-α were significantly higher in the DMBA-treated VDR^ΔIEC^ mice than in the VDR^loxp^ mice (Figure 4e). More universal bacteria were found in the breast tumors of VDR^ΔIEC^ mice by FISH staining (Figure 4f), suggesting an enhancement of the local bacteria within the breast. In the breast tissue of cancer patients, a variety of bacteria, i.e., *Streptococcus pyogenes*, *Streptococcus mitis*, *Lactobacillus, Methylobacterium*, and *Atopobium*, have been found in greater abundance in cancer tissue than in corresponding normal tissue^50,51^. We then used the FISH probe Strc 493 (for *Streptococcus spp.)* and found more *Streptococcus* bacteria in breast tumors of VDR^ΔIEC^ mice than in those of VDR^loxp^ mice, suggesting enhanced *Streptococcus spp*. within VDR^ΔIEC^ breast cancer tissue.

## Butyrate treatment reduced the breast tumor number, increased breast VDR expression, decreased proliferation, and increased apoptosis in VDR^ΔIEC^ mice

**Figure 5.**
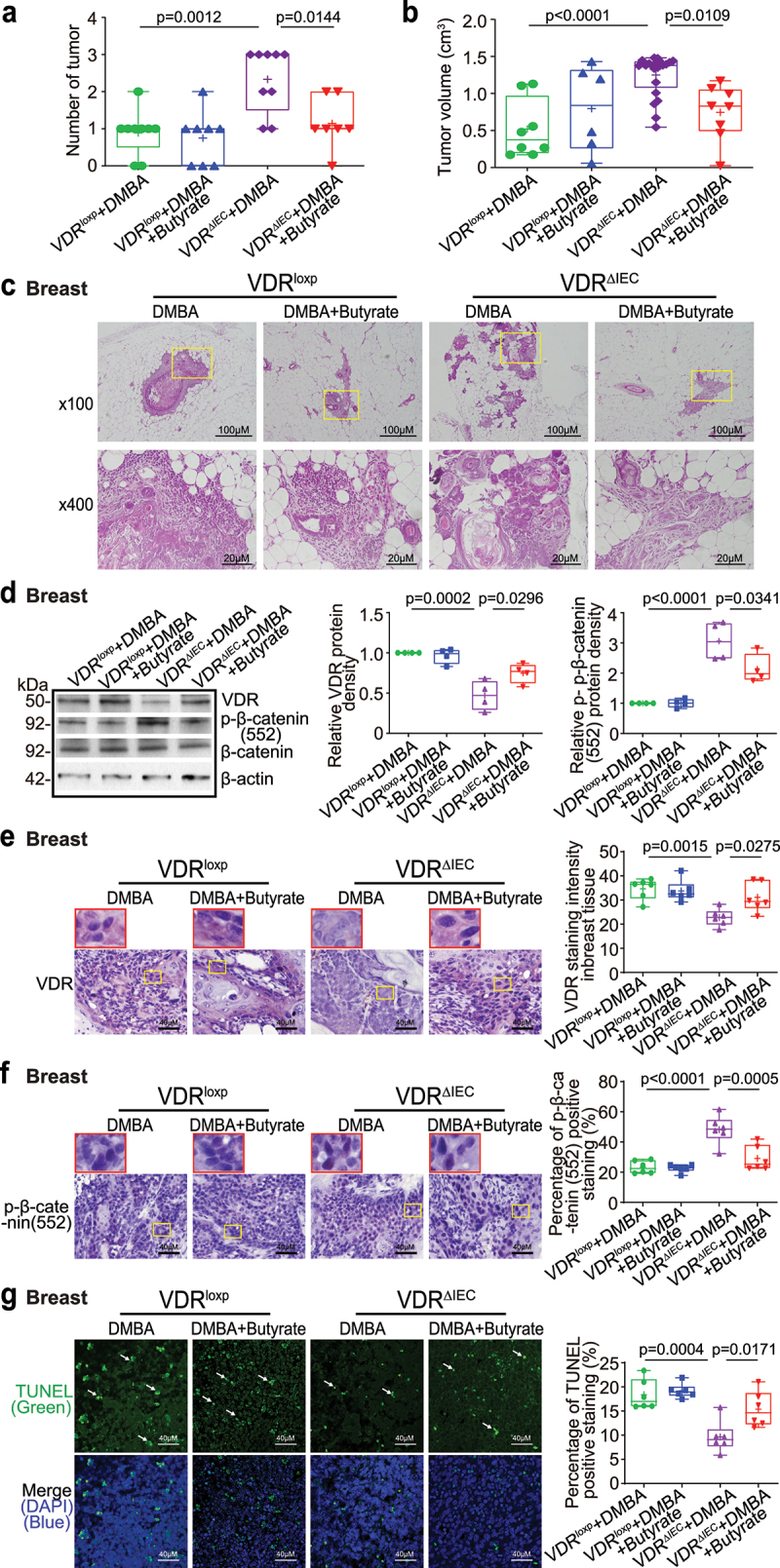
Butyrate-treated VDRΔIEC mice had fewer and smaller tumors, increased breast VDR expression, decreased breast *p*-β-catenin (552) expression, and increased cell apoptosis. (a) the number of breast tumors significantly decreased in VDR^ΔIEC^mice treated with butyrate. Data are expressed as the mean ± SD. N = 7–9, one-way ANOVA. (b) the breast tumor volumes were significantly smaller in VDR^ΔIEC^mice treated with butyrate. Data are expressed as the mean ± SD. N = 7–9, one-way ANOVA. (c) Representative H&E staining of mammary glands from the indicated groups. Images are from a single experiment and are representative of 7–9 mice per group. (d) VDR expression increased, while *p*-β-catenin (Ser552) expression decreased in breast tumor tissue in VDR^ΔIEC^ mice treated with butyrate. Data are expressed as the mean ± SD; N = 4, one-way ANOVA. (e) VDR was increased in breast tumor tissue in VDR^ΔIEC^ mice treated with butyrate, as shown by IHC staining. Images are from a single experiment and are representative of 6 mice per group. Red boxes indicate the selected area at higher magnification. Data are expressed as the mean ± SD. N = 6, one-way ANOVA. (f) *P*-β-catenin (Ser552) expression decreased in breast tumor tissue in VDR^ΔIEC^ mice treated with butyrate, as shown by IHC staining. Images are from a single experiment and are representative of 6 mice per group. Red boxes indicate the selected area at higher magnification. Data are expressed as the mean ± SD. N = 6, one-way ANOVA. (g) Apoptosis-positive cells were decreased in the breast tumor tissue of VDR^ΔIEC^mice treated with butyrate, as shown by TUNEL staining. Images are from a single experiment and are representative of 6 mice per group. Data are expressed as the mean ± SD. N = 6, one-way ANOVA. All *p* values are shown in the figures.

Because butyrate synthesis-related transferase was decreased in the feces of VDR^ΔIEC^ mice compared to VDR^loxp^ mice, we then hypothesized that butyrate treatment in mice could reduce the formation of breast tumors. We also tested the role of butyrate because it is known to increase VDR expression in the intestine^11^. Female VDR^loxp^ and VDR^ΔIEC^ mice were treated with 2.5% butyrate in the drinking water starting at the age of 6–7 weeks and ending 18 weeks after the first DMBA treatment. The breast tumor number was significantly decreased in VDR^ΔIEC^ mice treated with butyrate (Figure 5a). The breast tumor volume was significantly smaller in VDR^ΔIEC^ mice treated with butyrate than in those without butyrate treatment (Figure 5b). Pathological analysis showed that the mammary glands were smaller in size in VDR^ΔIEC^ mice treated with butyrate (Figure 5c). Increased protein expressions of VDR and reduced *p*-β-catenin (552) were observed in breast tumors of VDR^ΔIEC^ mice treated with butyrate (Figure 5d). Increased VDR expression was confirmed by IHC staining of breast tumor tissue in VDR^ΔIEC^ mice treated with butyrate (Figure 5e). In VDR^ΔIEC^ mice treated with butyrate, we found significantly reduced *p*-β-catenin (Ser552) in breast tumors (Figure 5f). Apoptosis-positive cells were also significantly increased in the breast tumors of VDR^ΔIEC^ mice treated with butyrate, as shown by TUNEL staining (Figure 5g).

## Butyrate treatment enhanced intestinal TJs, corrected dysbiosis, and inhibited inflammation

**Figure 6.**
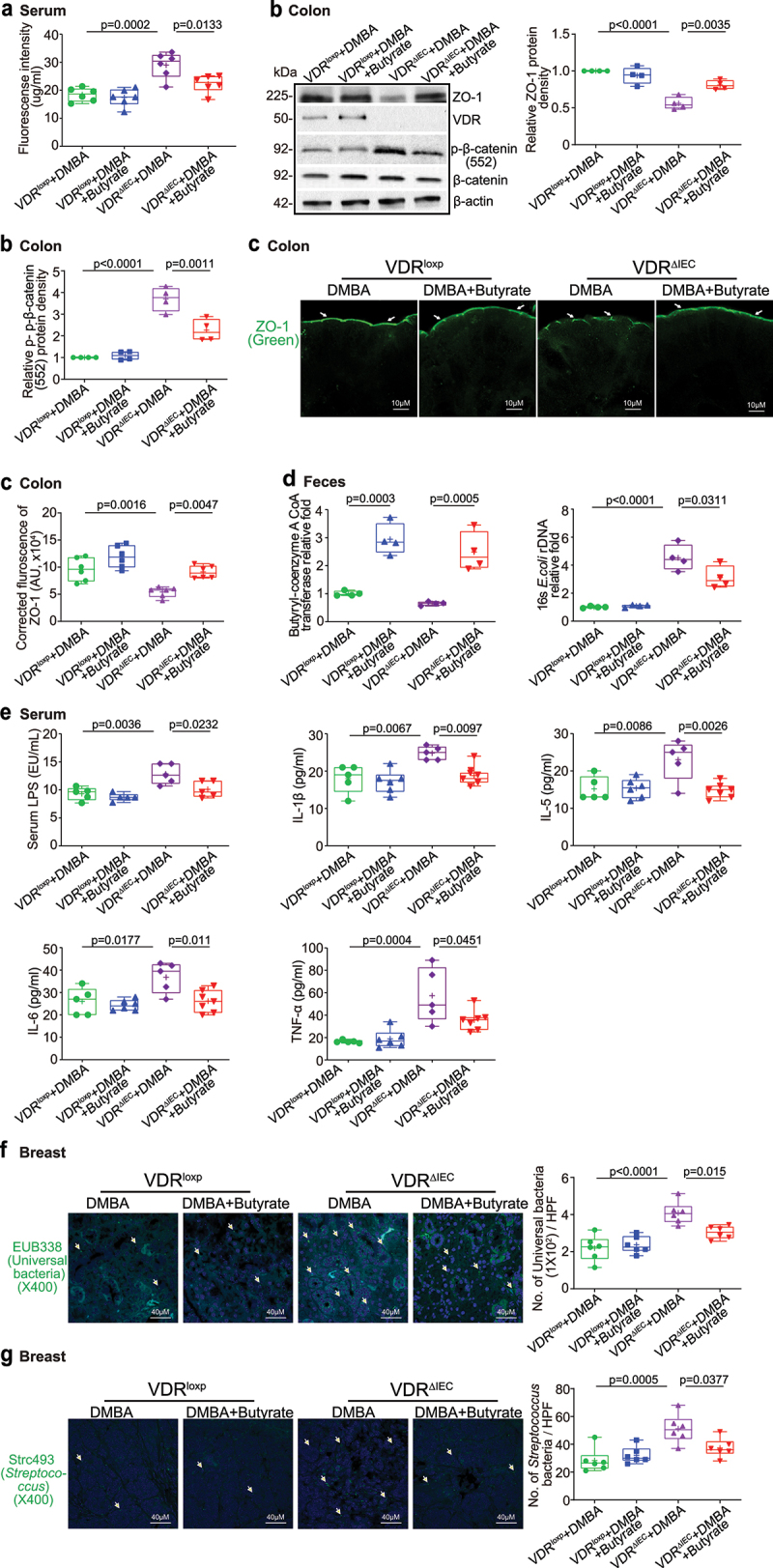
Butyrate treatment decreased intestinal permeability, increased intestinal ZO-1 expression, and decreased inflammation in VDR^ΔIEC^ mice. (a) Intestinal permeability decreased in VDR^ΔIEC^ mice treated with butyrate. Data are expressed as the mean ± SD. N = 6, one-way ANOVA. (b) ZO-1 expression increased and *p*-β-catenin (552) expression decreased in the intestine of VDR^ΔIEC^ mice treated with butyrate. Data are expressed as the mean ± SD. N = 4, one-way ANOVA. (c) ZO-1 expression increased in VDR^ΔIEC^ mice treated with butyrate, as determined by IF staining. Images are from a single experiment and are representative of 6 mice per group. Data are expressed as the mean ± SD. N = 6, one-way ANOVA. (d) Butyrate treatment increased butyryl-coenzyme a CoA transferase genes and decreased *E.* *coli* in the VDR^ΔIEC^ mice treated with butyrate. Data are expressed as the mean ± SD. N = 4, one-way ANOVA. (e) Butyrate treatment protected against increased inflammation in VDR^ΔIEC^ mice. Serum LPS, IL-1β, IL-5, IL-6, and TNF-α were significantly lower in VDR^ΔIEC^ mice treated with butyrate. Data are expressed as the mean ± SD. N = 5–7, one-way ANOVA. All *p* values are shown in the figures. (f) Less universal bacteria in breast tumor tissue of VDR^ΔIEC^ mice with butyrate treatment were found by fluorescence in situ hybridization. Images are from a single experiment and are representative of 6 mice per group. Data are expressed as the mean ± SD. N = 6, one-way ANOVA. All *p* values are shown in the figures. (g) Less *Streptococcus* bacteria in breast tumor tissue of VDR^ΔIEC^ mice with butyrate treatment were found by fluorescence in situ hybridization. Images are from a single experiment and are representative of 6 mice per group. Data are expressed as the mean ± SD. N = 6, one-way ANOVA. All *p* values are shown in the figures..

We found that intestinal permeability was decreased in VDR^ΔIEC^ mice treated with butyrate (Figure 6a). ZO-1 expression was increased in the colons of butyrate-treated VDR^ΔIEC^ mice, while *p*-β-catenin (552) expression was decreased in the colons of butyrate-treated VDR^ΔIEC^ mice (Figure 6b). Increased ZO-1 expression was confirmed by immunostaining of colon tissues from VDR^ΔIEC^ mice treated with butyrate (Figure 6c). There were an increase in the level of butyryl-CoA transferase gene and a decrease in *E. coli* in the feces of VDR^ΔIEC^ mice treated with butyrate (Figure 6d). Serum enterotoxin LPS and proinflammatory cytokines (i.e., IL-1β, IL-5, IL-6, and TNF-α) were significantly lower in VDR^ΔIEC^ mice treated with butyrate (Figure 6e), suggesting that butyrate protected mice from increased inflammation. FISH staining showed less universal bacteria and less *Streptococcus* bacteria in the breast tumor tissues of VDR^ΔIEC^ mice treated with butyrate (Figure 6f–g). Taken together, these data indicate that butyrate treatment led to a significant reduction in breast tumors, enhanced tight junctions, restored the microbiome, and inhibited inflammation in VDR^ΔIEC^ mice.

## Probiotics reduced breast tumors, increased breast VDR expression, decreased proliferation, and increased apoptosis in VDR^ΔIEC^ mice

**Figure 7.**
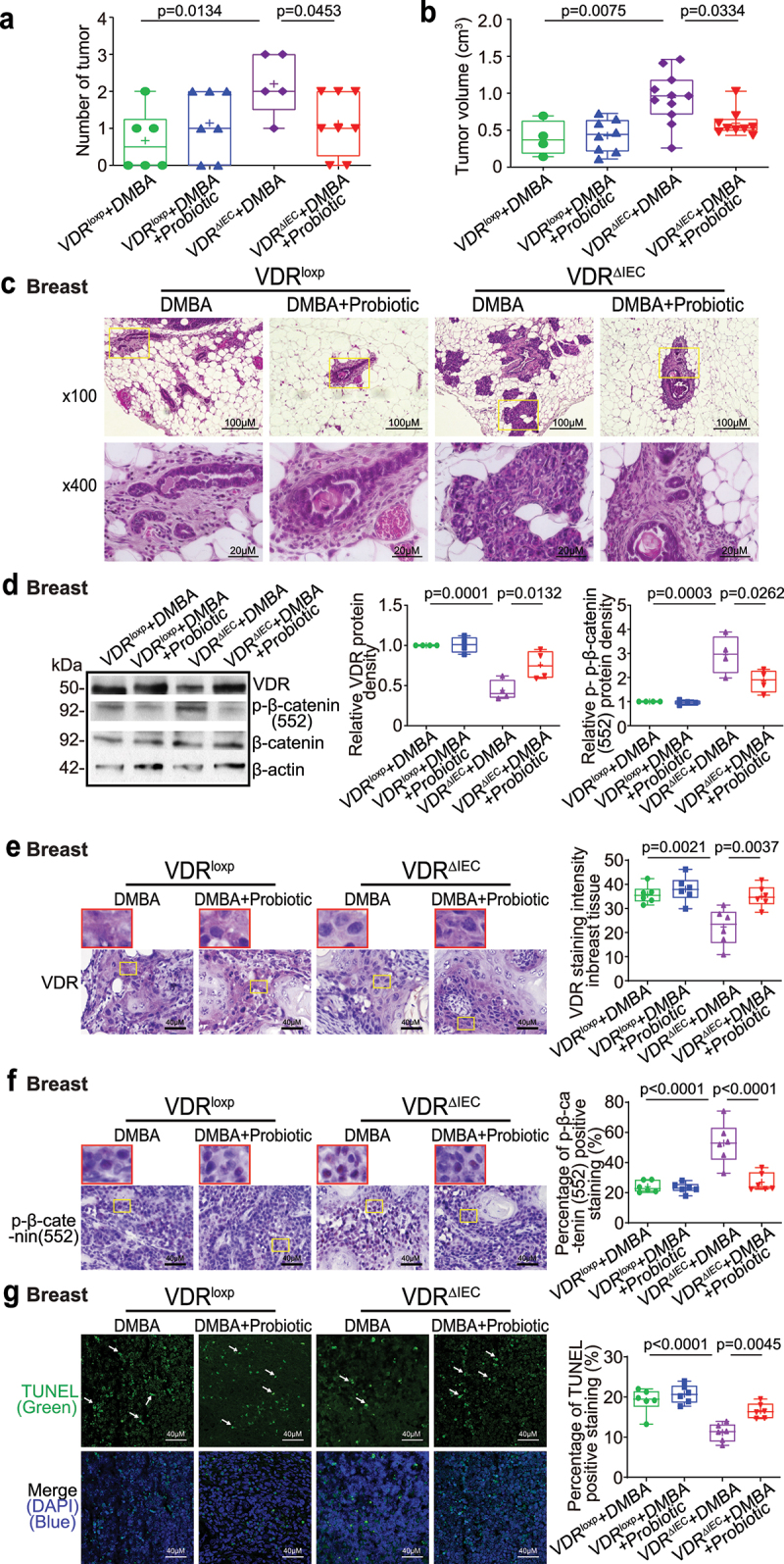
Probiotic-treated VDR^ΔIEC^ mice have fewer and smaller tumors, increased breast VDR expression, decreased expression of *p*-β-catenin (552), and increased cell apoptosis. (a) the number of breast tumors significantly decreased in the probiotic-treated VDR^ΔIEC^ mice. Data are expressed as the mean ± SD. N = 5–8, unpaired t test. (b) the volume of breast tumors was significantly smaller in the probiotic-treated VDR^ΔIEC^ mice. Data are expressed as the mean ± SD. N = 5–8, one-way ANOVA. (c) Representative H&E staining of mammary glands from the indicated groups. Images are from a single experiment and are representative of 6–8 mice per group. (d) VDR expression was increased, while *p*-β-catenin (552) expression was decreased in breast tumor tissue in the probiotic-treated VDR^ΔIEC^ mice. Data are expressed as the mean ± SD. N = 4, one-way ANOVA. (e) VDR was increased in breast tumor tissue in VDR^ΔIEC^ mice treated with probiotics, as shown by IHC staining. Images are from a single experiment and are representative of 6 mice per group. Red boxes indicate the selected area at higher magnification. Data are expressed as the mean ± SD. N = 6, one-way ANOVA. (f) *P*-β-catenin (552) expression decreased in breast tumor tissue in VDR^ΔIEC^ mice with probiotic treatment, as shown by IHC staining. Images are from a single experiment and are representative of 6 mice per group. Red boxes indicate the selected area at higher magnification. Data are expressed as the mean ± SD. N = 6, one-way ANOVA. (g) Apoptosis-positive cells were decreased in breast tumors of VDR^ΔIEC^ mice with probiotic treatment by TUNEL staining. Images are from a single experiment and are representative of 6 mice per group. Data are expressed as the mean ± SD. N = 6, one-way ANOVA. All *p* values are shown in the figures.

The probiotic *Lactobacillus plantarum* (*LP*) is known to increase the expression of intestinal VDR protein^52^. *LP* could promote the production of butyrate or other short-chain fatty acids (SCFAs)^53,54^. To test the role of probiotic treatment in breast cancer, female VDR^loxp^ and VDR^ΔIEC^ mice in the DMBA-probiotic-treated groups were gavaged daily with *LP* starting at 6–7 weeks of age and ending 18 weeks after the first DMBA treatment. The number of breast tumors significantly decreased in VDR^ΔIEC^ mice treated with probiotics (Figure 7a). The breast tumor volumes were significantly smaller in the VDR^ΔIEC^ mice with probiotic treatment (Figure 7b). Pathological analysis showed that mammary glands were smaller in size in the VDR^ΔIEC^ mice with probiotic treatment (Figure 7c). In the probiotic-treated VDR^ΔIEC^ mice, we found that probiotic treatment significantly restored the protein expression of VDR and reduced *p*-β-catenin (Ser552) in breast tumors (Figure 7d). Increased VDR expression was confirmed by the IHC staining of breast tumor tissues of VDR^ΔIEC^ mice with probiotic treatment (Figure 7e). Decreased *p*-β-catenin(552) expression was confirmed by IHC staining of breast tumor tissues from VDR^ΔIEC^ mice treated with butyrate (Figure 7f). By TUNEL staining, we found increased apoptotic cells in VDR^ΔIEC^ mice treated with butyrate (Figure 7g).

## Probiotics enhanced intestinal TJs and reduced inflammation in VDR^ΔIEC^ mice

**Figure 8.**
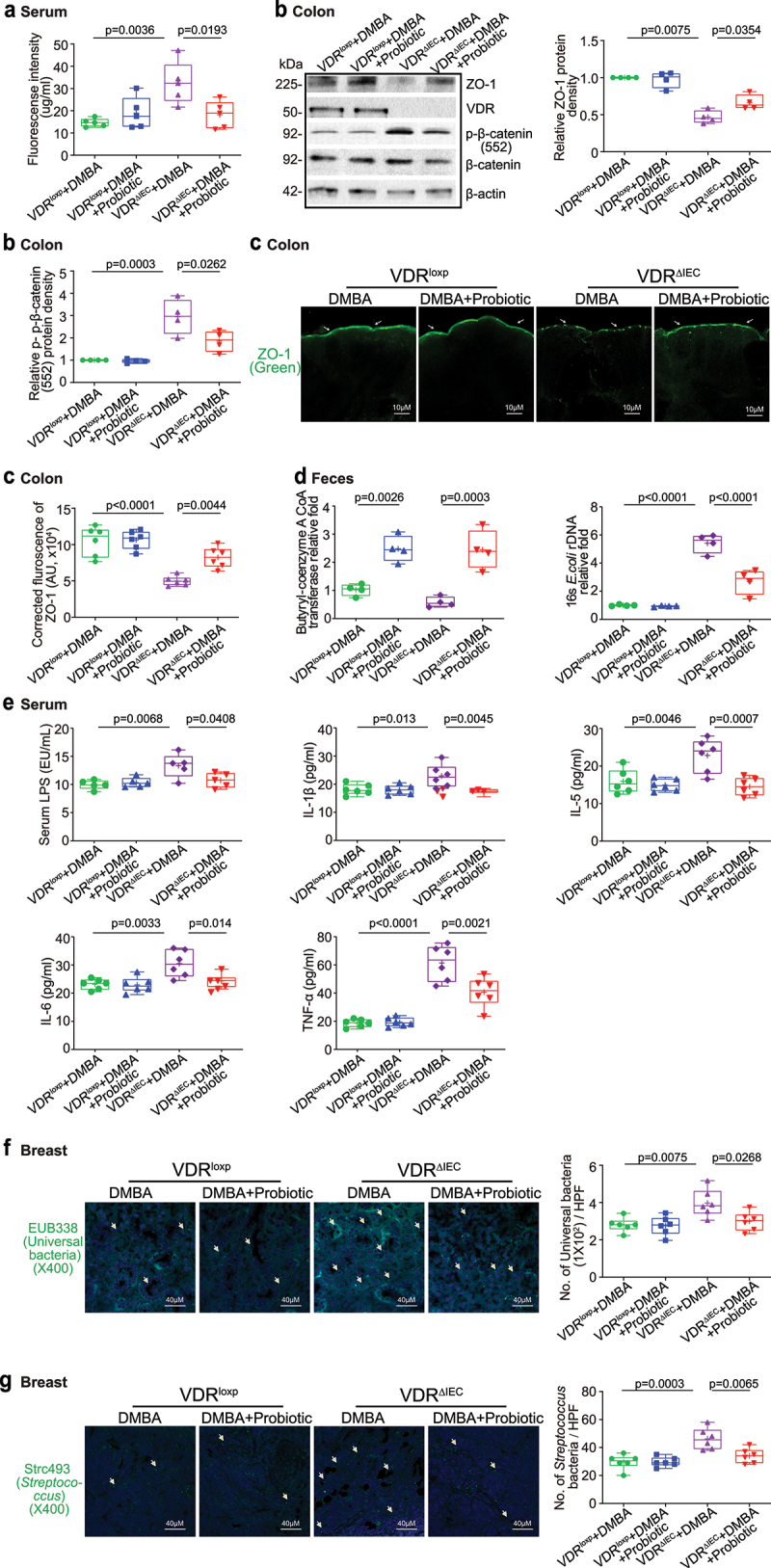
Probiotic-treated VDR^ΔIEC^ mice had decreased intestinal permeability, increased intestinal ZO-1 expression, and corrected dysbiosis and were protected against increased inflammation. (a) Intestinal permeability decreased in VDR^ΔIEC^ mice treated with probiotics. Data are expressed as the mean ± SD. N = 5, one-way ANOVA. (b) ZO-1 expression increased in the intestine of VDR^ΔIEC^ mice with probiotic treatment. Colonic *p*-β-catenin (552) expression decreased in the VDR^ΔIEC^ mice with probiotic treatment. Data are expressed as the mean ± SD. N = 4, one-way ANOVA. (c) ZO-1 expression increased in VDR^ΔIEC^ mice treated with probiotics, as shown by immunofluorescence staining. Images are from a single experiment and are representative of 6 mice per group. Data are expressed as the mean ± SD. N = 6, one-way ANOVA. (d) Probiotic treatment increased butyryl-CoA transferase genes and decreased *E.* *coli* in VDR^ΔIEC^ mice. Data are expressed as the mean ± SD. N = 4, one-way ANOVA. (e) Probiotic treatment protected against increased inflammation in VDR^ΔIEC^ mice. Serum LPS, IL-1β, IL-5, IL-6, and TNF-α were significantly lower in VDR^ΔIEC^ mice treated with probiotics. Data are expressed as the mean ± SD. N = 5–6, one-way ANOVA. All *p* values are shown in the figures. (f) Less universal bacteria in breast tumor tissue of VDR^ΔIEC^ mice with probiotic treatment were found by fluorescence in situ hybridization. Images are from a single experiment and are representative of 6 mice per group. Data are expressed as the mean ± SD. N = 6, one-way ANOVA. All *p* values are shown in the figures. (g) Less *Streptococcus* bacteria in breast tumor tissue of VDR^ΔIEC^ mice with probiotic treatment were found by fluorescence in situ hybridization. Images are from a single experiment and are representative of 6 mice per group. Data are expressed as the mean ± SD. N = 6, one-way ANOVA. All *p* values are shown in the figures.

We found that intestinal permeability decreased in VDR^ΔIEC^ mice treated with the probiotic *LP* (Figure 8a). ZO-1 expression increased in the colons of VDR^ΔIEC^ mice with probiotic treatment, whereas *p*-β-catenin (552) expression was decreased in the colons of probiotic-treated VDR^ΔIEC^ mice (Figure 8b). An increase in the level of colonic ZO-1 expression was confirmed by immunostaining in VDR^ΔIEC^ mice with probiotic treatment (Figure 8c). An increase in the butyryl-CoA transferase gene level and a decrease in *E. coli* were also observed in the VDR^ΔIEC^ mice treated with probiotic *LP* (Figure 8d). Moreover, serum LPS, IL-1β, IL-5, IL-6, and TNF-α were significantly lower in VDR^ΔIEC^ mice treated with probiotics (Figure 8e). We found fewer bacteria and *Streptococcus* in the breast tumor tissue of VDR^ΔIEC^ mice with probiotic treatment by FISH (Figure 8f–g). These data suggested that probiotic treatment might have several beneficial roles, e.g., reducing breast tumors, enhancing TJs, inhibiting inflammation, and restoring the microbiome, thus inhibiting tumorigenesis in VDR^ΔIEC^ mice.

## Discussion

Our current study fills the gaps in knowledge by revealing a previously unknown mechanism by which intestinal epithelial VDR is important for normal host homeostasis and protects against breast cancer. While the VDR^ΔIEC^ mice had normal VDR expression in the breast, the increased inflammation and bacterial loading/translocation still increased the risk of tumorigenesis in organs beyond the intestine. We identified increased intestinal permeability, chronic inflammation, and enhanced bacteria within breast tumors. Furthermore, by manipulating the gut microbiome using a beneficial microbial metabolite or probiotic bacteria, we were able to reduce the tumor burden in VDR^ΔIEC^ mice. Merging evidence has shown that enteric bacteria play a crucial role in the pathogenesis of breast cancer^36,55^. Our study further suggests new therapeutic targets for restoring intestinal VDR and microbiome functions in preventing breast cancer (see the Graphical Abstract). Clearly, research on intestinal VDR provides a framework to understand how intestinal dysfunction may inadvertently promote the development of distant cancer. It is an extension of VDR’s normal role in defense and repair. These insights are important for understanding health as well as disease. Multiple mechanisms by which VDR affects cancers have been found^6–9,56^. The 1,25D_3_/VDR complex might induce genes that suppress proliferation and maintain differentiation in normal mammary glands, and dysregulation of VDR signaling could predispose mammary epithelial cells to transformation^57,58^. However, the specific relationship between the function of intestinal VDR and the microbiome in breast tumorigenesis is not understood. An intricate symbiotic relationship has evolved between humans and microbes, especially the gut microbiota, which appear to influence the host at nearly every level and every organ system^36,55^. The microbiome, including the gut microbes and the microbiota at the breast site^59^, plays an important role in breast health and diseases^60^. However, little is known about the intestinal VDR regulation of the microbiome community in the gut and the breast. Our previous studies demonstrated that VDR KO mice are sensitive to bacterial invasion and exhibit severe damage of the intestine^11,16,17^. The VDR-bacterial interactions represent an example of a microorganism-induced program of host homeostasis. Dysregulation of bacterial-host interactions can result in chronic inflammation and overexuberant repair responses, and it is associated with the development of cancer^18–23^, not just in the intestine but also in other organs, e.g., the breast. Thus, our studies fill these gaps in knowledge by investigating the mechanisms by which intestinal epithelial VDR regulates the development of breast cancer.

We showed that the bacterial metabolite butyrate and probiotic treatment were able to reduce tumors in the breast *in vivo*. Dysbiosis in the breast can change the microenvironment, which causes mastitis and poses a potential risk of breast cancer. A recent study has further shown that breast tumor-resident microbiota, albeit at low biomass, play an important role in promoting metastasis^59^. Our study has shown the protective roles of lactic acid bacteria in breast tumorigenesis. Lactic acid bacteria are known for their beneficial health effects, including anticarcinogenic features. Different bacterial profiles in breast tissue exist between healthy women and those with breast cancer^61^. Patients with breast cancer had higher relative abundances of *Bacillus*, *Enterobacteriaceae*, and *Staphylococcus*. In VDR^ΔIEC^ mice, the abundance of butyrate-producing bacteria, e.g., Butyrivibrio fibrisolvens, is reduced^11^. Here, we further reported that the abundances of two beneficial bacterial species, *Lactobacillus johnsonii* and *Bifidobacterium pseudolongum*, were markedly downregulated in feces. There is known cross-feeding interplay between bifidobacteria and butyrate-producing bacteria^62^. These interactions possibly favor the coexistence of bifidobacterial strains with other bifidobacteria and with butyrate-producing bacteria in the colon^62^. Probiotic treatment helped to restore the healthy microbiome and its functions to produce beneficial metabolites. *Lactobacillus plantarum* promoted the production of butyrate or other SCFAs^53,54^ and increased VDR protein expression in the intestine^52^. We showed that *LP* treatment also increased bacterial butyryl-CoA CoA transferase expression.

Dysbiosis of the gut microbiome could indirectly participate in the development or progression of breast cancer via bacterial metabolites from the gut (SBA, SCFA, PAMPs, and vitamins) and immune and inflammatory modulators (TLRs and cytokines) that are inextricably interlinked with the microbiome from the GI tract^36^. VDR expression increases epithelial integrity and attenuates inflammation^63^. Interestingly, we found that some butyrate-related modules were also downregulated in VDR^ΔIEC^ mice. It has been reported that butyrate can induce growth arrest, apoptosis or differentiation in breast cancer cell lines^64^. We found that butyrate treatment in VDR^ΔIEC^ mice helped the hosts not only correct dysbiosis but also inhibit inflammatory cytokines, thus reducing breast tumors. Because low-dose proinflammatory cytokines are sufficient to induce bacterial endocytosis by epithelial cells, subclinical or low-grade changes may tip the balance of tolerance toward full blown inflammation owing to subsequent intracellular microbial sensing and paracellular permeability damage. With the current knowledge of the microbiome in the development of various cancers, understanding the interactions among the epithelium, microbiome, and metabolites could help in developing strategies for managing chronic inflammation in diseases, including cancers.

There are several limitations in our current study. Intestinal VDR KO might lead to hypocalcemia and weight loss unless mice were reared and maintained on a “rescue” diet high in calcium. In the current study, we used regular diet for all mice, not the diet high in calcium. Because we focused on the role of probiotics or a microbial metabolite in tumorigenesis, we provided the same regular food to the intestinal epithelial VDR KO mice in experimental groups (e.g., with or without probiotic treatment, with or without butyrate). The overall outcome of probiotic treatment or butyrate treatment significantly reduced the breast cancer tumorigenesis, regardless of the potential hypocalcemia in the KO mice. The role of “rescue” diet in the development of breast cancer will be tested in the future. Low VDR expression and diminished vitamin D/VDR signaling are observed in breast cancer^26,57^, and VDR might serve as a negative growth regulator of estrogen receptor (ER)-positive and ER-negative breast cancer cells^57,58^. However, the status of VDR may not be universally reduced in breast cancer. We did not have related human data on VDR protein levels in the intestine and breast. The current study only tested the DMBA-induced cancer model. Studies are needed to test the roles of butyrate and probiotics in other breast cancer models and human trials. Further study is needed to show whether microbiota transplants can affect breast tumors in intestinal VDR-deficient mice that were previously germ-free.

Epidemiological and experimental studies have indicated a protective action of vitamin D against cancer^65–71^. Vitamin D_3_ exerts its chemopreventive activity by interrupting the crosstalk between tumor epithelial cells and the tumor microenvironment in a VDR-dependent manner^67^. Moreover, there is increasing interest in using vitamin D compounds for disease prevention and therapy^72,73^. Maintaining intestinal VDR functions and a healthy gut microbiome will promote breast health. Our current study provides insights into alternative methods of enhancing VDR with the bacterial product butyrate and probiotics, thus reducing the risk of breast tumors.

In conclusion, our study has demonstrated that intestinal epithelial VDR deficiency significantly influences intestinal barrier function, microbiome profile/location, and breast tumorigenesis. Gut-breast-microbiome interactions indicate a new target for preventing and treating breast cancer. It could potentially open a direction in understanding the microbial-VDR interactions in breast diseases and developing a new protocol for risk assessment and prevention of extraintestinal illness.

## Materials and methods

### Animals

The intestine-specific VDR knockout VDR^ΔIEC^ mice were obtained by crossing the VDR^loxp^ mice with villin-cre mice (Jackson Laboratory, 004586), as we previously reported^11,74,75^. We further backcrossed this strain with C57BL/6 mice for more than 10 generations after arriving at our animal facility. Experiments were performed on 6- to 7-week-old female mice. Mice were provided with water ad libitum and were maintained on a 12-h dark/light cycle. Mice had unrestricted access to Teklad Irradiated Diet 7912 (Envigo, Madison, WI, USA). The animal work was approved by the UIC Office of Animal Care. The animal protocol numbers used in this study are ACC 16–180, ACC 19–139, and ACC 20-058.

## Induction of breast cancer by DMBA in mice

Female VDR^loxp^ and VDR^ΔIEC^ mice (6–7 weeks old) were randomly assigned to either the control or DMBA groups. Mice were administered a weekly dose of 1.0 mg of DMBA (Sigma‒Aldrich, Milwaukee, WI, USA) in 0.2 ml of corn oil or an equal volume of corn oil alone (vehicle) by oral gavage. The DMBA treatment lasted for 6 weeks. Then, the mice were mated continuously to provide an oscillating hormonal environment and monitored until tumor development. Starting at 12 weeks of age, mice were examined for mammary tumors twice a week. The tumor volume (V) was calculated with caliper measurements using the formula V= (W^2^ × L)/2, where V is the tumor volume, W is the tumor width, and L is the tumor length^76^. The mice were sacrificed under anesthesia at 18 weeks after the first DMBA treatment or at the time when palpable mammary tumors reached a volume of 2 cm^3^. Tumor counts and measurements were performed in a blinded fashion under a stereo-dissecting microscope (Nikon SMZ1000, Melville, NY, USA).

Eighteen weeks after DMBA/butyrate treatment, we used the mice for the intestinal permeability study. Fluorescein dextran (molecular weight 4 kDa, diluted in HBSS) was gavaged (50 mg/kg mouse) 4 hours before sample harvest. Mice were anesthetized with isoflurane; the depth of anesthesia was assessed with a toe pinch, and then blood was collected via cardiac puncture followed by cervical dislocation. Mouse blood samples were collected for the intestinal permeability test. Following euthanasia, GI tissues were collected for H&E staining, western blotting, and immunofluorescence staining.

## Butyrate treatment in mice

Female VDR^loxp^ and VDR^ΔIEC^ mice (6–7 weeks old) were randomly assigned to either the DMBA alone or DMBA-butyrate groups. The DMBA control group received filtered drinking water without sodium butyrate. The DMBA-butyrate-treated group received 2.5% sodium butyrate (Sigma‒Aldrich, Milwaukee, WI, USA) in filtered drinking water. Starting at 12 weeks of age, mice were examined for mammary tumors twice a week. The mice were sacrificed under anesthesia at week 18 post-butyrate treatment or at the time when palpable mammary tumors reached a volume of 2 cm^3^.

## Probiotic treatment in mice

Female VDR^loxp^ and VDR^ΔIEC^ mice (6–7 weeks old) were randomly assigned to either the DMBA alone or DMBA-probiotic groups. Mice were gavaged daily with *Lactobacillus plantarum* (1 × 10^7^ CFU) in 0.1 ml of HBSS or an equal volume of HBSS. Starting at 12 weeks of age, mice were examined for mammary tumors twice a week. The mice were sacrificed under anesthesia at week 18 after the first DMBA treatment or at the time when palpable mammary tumors reached a volume of 2 cm^3^.

## Intestinal permeability

Fluorescein dextran (molecular weight 4 kDa, diluted in HBSS) was gavaged (50 mg/kg mouse) 18 weeks after the first DMBA treatment. Four hours later, mouse blood samples were collected for fluorescence intensity measurement, as previously reported^77^.

## Hematoxylin and eosin staining

Slides containing mouse colon (proximal or distal colon) sections (5 μm) were deparaffinized in xylene and passed through graded alcohol. They were then stained with hematoxylin and eosin following a previously described method^74^.

## Western blot analysis and antibodies

Mammary tumors and grossly normal mammary glands from parous age-matched control mice were excised, and portions of the tissues were prepared for western blotting. Mouse colonic epithelial cells were collected by scraping the tissue from the colon of the mouse, including the proximal and distal regions. The cells were sonicated in lysis buffer (10 mM Tris, pH 7.4, 150 mM NaCl, 1 mM EDTA, 1 mM EGTA, pH 8.0, and 1% Triton X-100) with 0.2 mM sodium orthovanadate and protease inhibitor cocktail. The protein concentration was measured using Bio-Rad Reagent (Bio-Rad, Hercules, CA, USA) and then sonicated. Equal amounts of protein were separated by SDS-polyacrylamide gel electrophoresis, transferred to nitrocellulose membranes, and immunoblotted with primary antibodies. The following antibodies were used: anti-ZO-1 (Invitrogen, 33–9100, Carlsbad, CA, USA), anti-p-β-catenin (552) (Cell Signaling, 9566, Danvers, MA, USA), anti-β-catenin (BD Biosciences, Franklin Lakes, NJ, USA), anti-VDR (Santa Cruz Biotechnology, SC-13133, Dallas, TX, USA), and anti-β-actin (Sigma‒Aldrich, A5316, St. Louis, MO, USA) antibodies and were visualized by ECL (Thermo Fisher Scientific, 32106, Waltham, MA, USA). Membranes that were probed with more than one antibody were stripped before reprobing. Quantity One software was used for the quantification of the western blot bands. Briefly, the “rectangular tool” was first selected to measure the background and the bands of western blots one by one. The “density” and “volume” values after measurement were transferred to an Excel file. With the subtraction of background measurement, the “density” values for each band on the western blot were calculated.

## Immunofluorescence

Colonic or breast tissues were freshly isolated and embedded in paraffin wax after fixation with 10% neutral buffered formalin. Immunofluorescence was performed on paraffin-embedded sections (4 μm) after preparation of the slides as described previously^78^ followed by incubation for 1 hour in blocking solution (2% bovine serum albumin, 1% goat serum in HBSS) to reduce nonspecific background staining. The tissue samples were incubated overnight with the primary anti-ZO-1 antibody at 4°C. Slides were washed 3 times for 5 minutes each at room temperature in wash buffer. Samples were then incubated with the secondary antibody (goat anti-rabbit Alexa Fluor 488, Molecular Probes, CA; 1:200) for 1 hour at room temperature. Tissues were mounted with a SlowFade Antifade Kit (Life Technologies, s2828, Grand Island, NY, USA), followed by a coverslip, and the edges were sealed to prevent drying. Specimens were examined with a Zeiss laser scanning microscope LSM 710 (Carl Zeiss Inc., Oberkochen, Germany).

## Immunohistochemistry (IHC)

After preparation of the slides, antigen retrieval was achieved by incubating the slides for 15 min in preheated sodium citrate (pH 6.0) buffer followed by 30 min of cooling at room temperature. Endogenous peroxidases were quenched by incubating the slides in 3% hydrogen peroxide for 10 min, followed by three rinses with HBSS, and incubation for 1 hour in 3% BSA+1% goat serum in HBSS to reduce nonspecific background. Primary antibodies against VDR or *p*-β-catenin (552) were applied overnight in a cold room. After three rinses with HBSS, the slides were incubated in secondary antibody (1:100, Jackson ImmunoResearch Laboratories, Cat. No. 115-065-174, West Grove, PA, USA) for 1 hour at room temperature. After washing with HBSS for 10 minutes, the slides were incubated with vectastain ABC reagent (Vector Laboratories, Cat. PK-6100, Burlingame, CA 94,010, USA) for 1 hour. After washing with HBSS for five minutes, color development was achieved by applying a peroxidase substrate kit (Vector Laboratories, Cat. No. SK-4800, Burlingame, CA 94,010) for 2 to 5 minutes, depending on the primary antibody. The duration of peroxidase substrate incubation was determined through pilot experiments and was then held constant for all of the slides. After washing in distilled water, the sections were counterstained with hematoxylin (Leica, Cat. No. 3801570, Wetzlar, Germany), dehydrated through ethanol and xylene, and cover‐slipped using Permount (Fisher Scientific, Cat. No. SP15–100, Waltham, MA, USA).

## Terminal deoxynucleotidyl transferase dUTP nick end labeling (TUNEL) staining

The number of apoptotic cells was determined using the *In Situ* Cell Death Detection Kit (Sigma‒Aldrich, 11684795910, St. Louis, MO, USA) on paraffin-embedded tissue sections. Briefly, antigen retrieval was achieved after deparaffination and rehydration by incubating the slides for 15 min in preheated sodium citrate (pH 6.0) buffer. Then, the slides were washed with PBS for 10 minutes. After blocking, the slides were incubated with a TUNEL Reaction Mixture for 1 hour at 37°C. The tissues were mounted with a SlowFade Antifade Kit (Life Technologies, s2828, Grand Island, NY, USA) after a 10-minute wash with PBS. The staining was examined with a Zeiss laser scanning microscope LSM 710 (Carl Zeiss Inc., Oberkochen, Germany).

## Real-time PCR measurement of bacterial DNA

DNA was extracted from mouse feces using the EZNA Stool DNA Kit (Omega Biotek, Inc. D4015–01, Norcross, GA 30,071). Quantitative real-time PCR was conducted using the CFX96 Real-time PCR detection system (Bio-Rad Laboratories, Hercules, CA, USA) and iTaq^TM^ Universal SYBR green supermix (Bio-Rad Laboratories, 1725121, Hercules, CA, USA) according to the manufacturer’s directions. All expression levels were normalized to universal bacteria levels of the same sample. The percent expression was calculated as the ratio of the normalized value of each sample to that of the corresponding untreated control cells. All real-time PCRs were performed in triplicate. Primer sequences were designed using Primer-BLAST or were obtained from Primer Bank primer pairs listed in Table 1.

**Table 1.** Real-time PCR Primers.

Primer Name	Sequence
Butyryl-CoA transferase F	5’-GCIGAICATTTCACITGGAAYWSITGGCAYATG-3’
Butyryl-CoA transferase R	5’-CCTGCCTTTGCAATRTCIACRAANGC-3’
*E. coli* F	5’-CCTACGGGAGGCAGCAGT-3’
*E. coli* R	5′-CGTTTACGGCGTGGACTAC-3′
*Butyrivibrio fibrisolvens F*	5’-CTAACACATGCAAGTCGAACG-3’
*Butyrivibrio fibrisolvens R*	5’-CCGTGTCTCAGTCCCAATG-3’
Universal bacteria F	5’-TCCTACGGGAGGCAGCAGT-3’
Universal bacteria R	5’-GGACTACCAGGGTATCTAATCCTGTT-3’

## Multiplex ELISA

Mouse blood samples were collected by cardiac puncture and placed in tubes containing EDTA (10 mg/mL). Mouse cytokines were measured using a Cytokine & Chemokine Convenience 26-Plex Mouse ProcartaPlex™ Panel 1 (Invitrogen, EPXR260 -26,088-90, Carlsbad, CA) according to the manufacturer’s instructions. Briefly, beads of defined spectral properties were conjugated to protein-specific capture antibodies and added along with samples (including standards of known protein concentration, control samples, and test samples) into the wells of a filter-bottom microplate, where proteins bound to the capture antibodies over the course of a 2-hour incubation. After washing the beads, protein-specific biotinylated detector antibodies were added and incubated with the beads for 1 hour. After removal of excess biotinylated detector antibodies, the streptavidin-conjugated fluorescent protein R-phycoerythrin was added and allowed to incubate for 30 minutes. After washing to remove unbound streptavidin – R-phycoerythrin, the beads were analyzed with the Luminex detection system (Bio-Rad, Bio-Plex 200 Systems, Hercules, CA).

## Serum LPS detection

LPS in serum samples was measured with limulus amebocyte lysate chromogenic end point assays (Hycult Biotech, HIT302, Plymouth, PA) according to the manufacturer’s instructions. The samples were diluted 1:4 with endotoxin-free water and then heated at 75°C for 5 minutes on a warm plate to denature the protein before the reaction. A standard curve was generated and used to calculate the concentrations, which were expressed as EU/mL, in the serum samples.

## Shotgun metagenomic sequencing and bioinformatics analysis

### Study design and sampling

VDR^ΔIEC^ and control VDR^LoxP^ mouse strains were used in this study (males and females; aged 6 to 8 weeks). The breeders were set up at a similar time to obtain enough knockout and control mice of a similar age for sample collection. The mice used in this study were littermates, and they were transferred to different cages after weaning. All the animals used in this study were housed in the same room of the Biologic Resources Laboratory (BRL) at the University of Illinois at Chicago following the UIC Animal Care Committee’s Animal Care Policy. All mice for VDR^LoxP^ (males *n* = 3 and females *n* = 7) and VDR^ΔIEC^ (males *n* = 5 and females *n* = 5) were randomly assigned to each group.

Many methods are used to maintain biosecurity within the UIC animal facilities. First, the health monitoring program for mice utilizes sentinel animals to assess the pathogen status of UIC colonies. Sentinel mice are tested quarterly by serology, PCR, and parasitology. Comprehensive serologic testing of sentinel mice is performed annually. Second, animal housing measures are used to control the spread of infectious agents. The mice are housed in sterilized static microisolator cages, which provide an effective barrier to the entry and spread of microbial agents. Another means to decrease pathogen exposure and spread is through disinfection of all shared-use equipment and space prior to use. Doing so helps to ensure animal health, which in turn minimizes confounding variables to research models and maintains the specific pathogen-free status of animal colonies.

Fecal sample collection and shotgun metagenomic sequencing

Fresh fecal samples were collected and placed into sterile tubes with dry ice and sent to the University of Illinois at Chicago Research Resources Center for genomic sequencing. DNA from the samples was extracted with a DNeasy Power Fecal Kit (Qiagen, Hilden, Germany) according to the manufacturer’s instructions with slight modification as described previously^13^. Shotgun metagenomic sequencing was performed with the Illumina HiSeq system as described in our previous publications^16,79^. After checking the quality, filtering the reads, removing noise sequences, and metagenomic assembly were performed^80^, the resulting assemblies were filtered. DNA reads shorter than 1,000 nucleotides were excluded and classified with Centrifuge^81^. Finally, each gene was taxonomically annotated by searching the comprehensive NCBI GenBank nonredundant nucleotide database.

## Statistical analysis

All data are expressed as the mean ± SD. All statistical tests were 2-sided. All *p* values<0.05 were considered statistically significant. Based on data distributions, the differences between samples were analyzed using Welch’s *t* test or unpaired *t* test for two groups and one-way ANOVA for more than two groups as appropriate. Statistical analyses were performed using GraphPad Prism 8 (GraphPad, Inc., San Diego, CA, USA).

## Supplementary Material

Supplemental Material
