# Supplementary material for: Intestinal vitamin D receptor protects against extraintestinal breast cancer tumorigenesis
Source: Gut Microbes. 2023 Apr 19;15(1):2202593. doi: 10.1080/19490976.2023.2202593 (PMC10120454; doi:10.1080/19490976.2023.2202593)
Supplement: Supplemental Material [file KGMI_A_2202593_SM8595.pdf]

Supplementary Figure 1. The morphology of ileum in VDR^{loxp} and VDR$^{\Delta IEC}$ mice treated with DMBA

Representative H&E staining of ileum from the indicated groups. Images were from a single experiment and are representative of 8-13 mice per group.

Supplementary Figure 2. The abundance of bacteria in VDR^{loxp} and VDR$^{\Delta IEC}$ mice

The abundance of *Desulfitobacterium hafniense* and *Faecalibacterium prausnitzii* that could synthesize the butyryl-CoA CoA transferase and *E. coli*. Data are expressed as mean ± SD, Welch's *t*-test, n=10 each group.

S. Fig. 1

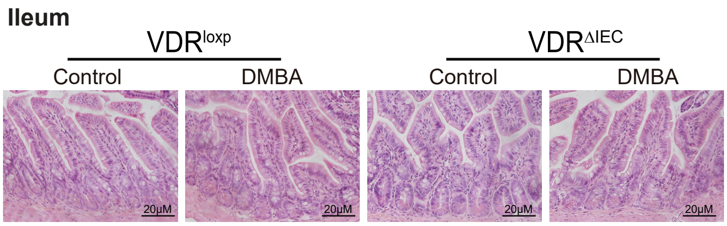
Ileum
VDR^loxp
VDR^ΔIEC
Control
DMBA
Control
DMBA
20μM
20μM
20μM
20μM

S. Fig. 2

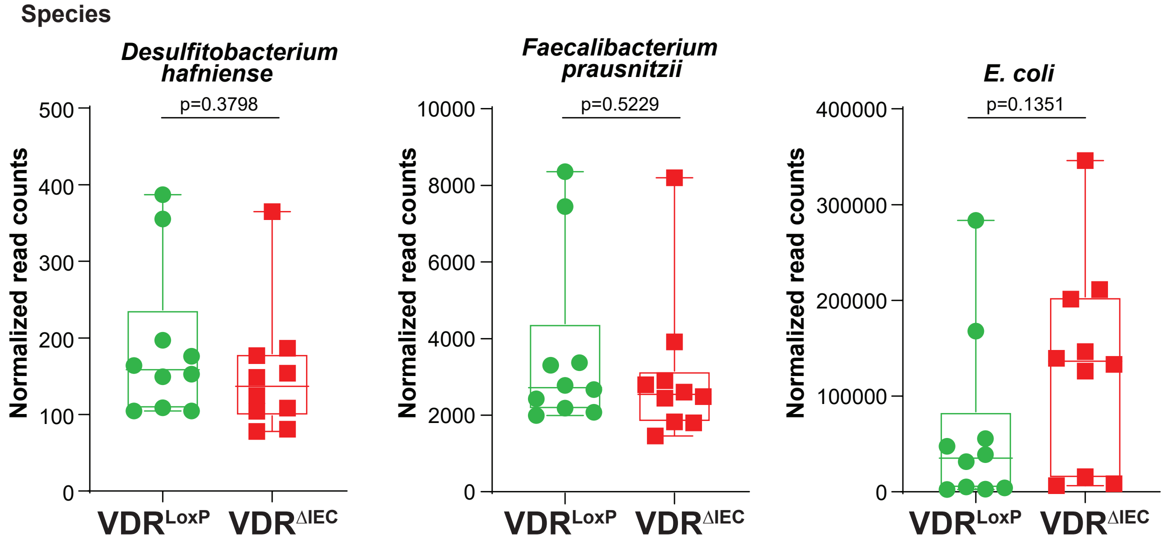

Species
Desulfitobacterium hafniense
p=0.3798
Faecalibacterium prausnitzii
p=0.5229
E. coli
p=0.1351
Normalized read counts
0
100
200
300
400
500
2000
4000
6000
8000
10000
100000
200000
300000
400000
VDRLoxP
VDRΔIEC
